# Editorial: Viral interactions with the nucleus, volume II

**DOI:** 10.3389/fmicb.2024.1419921

**Published:** 2024-05-10

**Authors:** Francesco Ciccarese, Xiaonan Zhang, Shadi Shahriari, Reena Ghildyal

**Affiliations:** ^1^Immunology and Molecular Oncology Unit, Veneto Institute of Oncology IOV-IRCCS, Padova, Italy; ^2^Centre for Research in Therapeutic Solutions, Faculty of Science and Technology, University of Canberra, Canberra, ACT, Australia

**Keywords:** nucleocytoplasmic trafficking, virus pathogenesis, virus assembly, virus-host interaction, DNA virus, RNA virus

Viruses are infective agents involved in a plethora of pathologies, ranging from the common cold such as Rhinovirus, to lethal diseases, such as Ebola. In recent times, the world has weathered crises caused by SARS-CoV-2 that killed millions of people worldwide. The still evolving economic and social disruption caused by the pandemic has highlighted the potential medical, health and socioeconomic impacts of viral infections.

Despite the large variety of viral families, all viruses are intracellular pathogens that, upon release of their genetic material inside the host cell, hijack the cell replication machinery in their favor and can inhibit several antiviral mechanisms. The regulated transport of proteins between the nucleus and the cytoplasm through the nuclear pore complex plays a pivotal role in cell physiology. Thus, it is not surprising that many viruses impair or exploit the nucleocytoplasmic trafficking pathways to blunt antiviral responses or complete their replication in host cells.

Dissecting the interactions between viruses and nucleocytoplasmic trafficking was fundamental in understanding the pathways involved in nuclear import and export and their role in cell biology. Moreover, identifying the mechanisms through which viruses impact host cell nucleocytoplasmic trafficking pathways may be critical to a deeper comprehension of the pathophysiology of viral diseases. There is also evidence that oncogenic viruses exploit the nuclear transport pathways and that the alterations in nucleocytoplasmic trafficking observed in cancers with viral etiology could be associated with tumorigenesis and metastasis.

The overarching aim of this Research Topic was to uncover the central role of nucleocytoplasmic trafficking in viral pathogenesis. Within this topic, four research articles and a perspective have been published.

Some viral proteins use their ability to access the nucleus through a nuclear localization signal (NLS) to relocalize host cell proteins, which, in turn, could exploit their nuclear export signal (NES) to bring viral proteins out of the nucleus. In this context, Zhou et al. studied the interaction between the capsid (Cap) protein of porcine circovirus type 2 (PCV2) and the host protein DEAD-box RNA helicase 21 (DDX21). The NLS of Cap directly interacts with the C-terminal domain of DDX21, relocalizing it from the nucleolus to the nucleus in the early phase (until 48 h) of viral infection. Silencing of DDX21 inhibited, while DDX21 overexpression enhanced PCV2 replication in infected cells (which takes place in the nucleus), indicating that nuclear relocalization of DDX21 is necessary for viral replication. DDX21 protein is also involved in antiviral innate immune responses. Interestingly, Zhou et al. observed that at 72 h post-infection, Cap relocalizes DDX21 to the cytoplasm, thus probably suppressing the antiviral response of infected cells.

Looking for human proteins that could interact with the non-structural protein 3C protease (3Cpro) of enterovirus 71 (EV71), a pathogen involved in hand, foot, and mouth disease, Li et al. found that 3Cpro interacts with and cleaves tumor necrosis factor receptor-associated factor 3 (TRAF3) interacting protein 3 (TRAF3IP3), a host protein that is involved in restricting the replication of EV71. 3Cpro has an NLS that brings it into the nucleus of infected cells, where it cleaves many host proteins including transcription factors involved in the antiviral response. Interestingly, TRAF3IP3, through its NES, relocates 3Cpro out of the nucleus, thus inhibiting EV71 replication by allowing the antiviral mechanisms of infected cells to continue.

The trafficking of viral proteins through the nucleus is functional in viral replication and suppression of the innate immune antiviral mechanisms. In some cases, the pathological role of nuclear import of viral proteins has yet to be discovered. Metzger et al. studied the subcellular localization of the non- structural polyprotein ORF1 of the hepatitis E virus (HEV), a major cause of acute hepatitis worldwide. Using confocal microscopy, they observed that ORF1 is mainly localized inside the nucleus and in perinuclear structures in the cytoplasm, where it colocalizes with the structural proteins ORF2 and ORF3. Moreover, using the RNAscope technology, they also detected the viral RNA in the same perinuclear substructures, thus identifying the putative viral factories of HEV.

Sattar et al. discovered that the spike (S) protein of SARS-CoV-2 contains an NLS and can be translocated into the nucleus of infected cells, along with its mRNA (or, possibly, the entire viral genome). Strikingly, the NLS of SARS-CoV-2 S protein derives from the fourth genomic insertion that arose in the S gene of SARS-CoV-2 and is thus unique in the *Coronaviridae* family. The role of the nuclear translocation of S protein and whether it contributes to immune evasion is yet to be determined.

The disruption of nucleocytoplasmic trafficking could account for the clinical manifestations observed in some diseases with viral etiology. Moorhouse et al. reviewed the role of human rhinoviruses (RV) in cleavage of nucleoporin (Nup) 153 and airway remodeling. Severe RV infection in early childhood is associated with development of asthma. Airway remodeling comprises several structural changes to the airway wall, leading to a reduction in air passage through the airway, and is present in almost all asthmatic cases. Among the features of airway remodeling, the epithelial-to- mesenchymal transition (EMT) and the fibroblast-to-myofibroblast transition (FMT) play a significant role in the pathogenesis of asthma. Both EMT and FMT are promoted by TGF-β, whose levels are high in the airways of asthmatic patients. Moorhouse et al. hypothesized that, through the cleavage of Nup153, the proteases 2A and 3C of RV disrupt the nucleocytoplasmic trafficking of proteins, thus inducing retention of SMAD2 in the nucleus and exacerbating TGF-β signaling, resulting in airway remodeling.

In conclusion, there is evidence that some viruses can impinge on nucleocytoplasmic trafficking of host cells, leading to a subversion of cell physiology that is functional for viral replication and evasion from the host innate immune response and can explain the pathophysiology of viral diseases.

## Author contributions

FC: Writing – original draft. XZ: Writing – review & editing. SS: Writing – review & editing. RG: Writing – review & editing.

